# Principal-component-based multivariate regression for genetic association studies of metabolic syndrome components

**DOI:** 10.1186/1471-2156-11-100

**Published:** 2010-11-09

**Authors:** Hao Mei, Wei Chen, Andrew Dellinger, Jiang He, Meng Wang, Canddy Yau, Sathanur R Srinivasan, Gerald S Berenson

**Affiliations:** 1 Epidemiology Department, School of Public Health and Tropical Medicine, Tulane University, New Orleans, USA; 2 Tulane Center for Cardiovascular Health, Tulane University Health Sciences Center, New Orleans, USA; 3 Center for Human Genetics, Duke University, Durham, NC, USA; 4 School of Life Science, Nanjing University, Nanjing, PR China; 5 Biostatistics Department, School of Public Health and Tropical Medicine, Tulane University, New Orleans, USA

## Abstract

**Background:**

Quantitative traits often underlie risk for complex diseases. For example, weight and body mass index (BMI) underlie the human abdominal obesity-metabolic syndrome. Many attempts have been made to identify quantitative trait loci (QTL) over the past decade, including association studies. However, a single QTL is often capable of affecting multiple traits, a quality known as gene pleiotropy. Gene pleiotropy may therefore cause a loss of power in association studies focused only on a single trait, whether based on single or multiple markers.

**Results:**

We propose using principal-component-based multivariate regression (PCBMR) to test for gene pleiotropy with comprehensive evaluation. This method generates one or more independent canonical variables based on the principal components of original traits and conducts a multivariate regression to test for association with these new variables. Systematic simulation studies have shown that PCBMR has great power. PCBMR-based pleiotropic association studies of abdominal obesity-metabolic syndrome and its possible linkage to chromosomal band 3q27 identified 11 susceptibility genes with significant associations. Whereas some of these genes had been previously reported to be associated with metabolic traits, others had never been identified as metabolism-associated genes.

**Conclusions:**

PCBMR is a computationally efficient and powerful test for gene pleiotropy. Application of PCBMR to abdominal obesity-metabolic syndrome indicated the existence of gene pleiotropy affecting this syndrome.

## Background

Quantitative traits often underlie increased risk for complex diseases. To understand the genetic basis of such traits, each trait is often separately tested for association with one or more markers. This approach has two disadvantages: 1) independent tests of each trait may lead to issues related to multiple testing; and 2) if a locus affects two or more traits, a single-trait study may lose the power to detect a pleiotropic effect, where a single gene influences multiple phenotypic traits.

In the past decade, simultaneous analysis of multiple traits in the context of linkage mapping of quantitative trait loci (QTL) has attracted much attention. Three approaches to simultaneous analysis have been developed and broadly applied, the first of which is generalization of maximum likelihood (ML) [1,2]. Although this method can be applied to multiple traits, a large number of correlated traits requires the simultaneous estimation of too many parameters, restraining its practical use [3]. The second approach, first proposed by Haley & Knott, is multivariate regression [4-7]. This approach is computationally faster than maximum likelihood and is available in most statistical software packages. But as with the ML method, the requirement for simultaneous estimates of a large number of parameters may limit its application. The third approach is based on transformation of original traits to a reduced number of canonical variables [3,8]. This approach is often implemented in two steps. First, principal components of original traits are identified to generate canonical variables. Next, a classical single trait method is used as the test of linkage between a candidate locus and a canonical variable. The test is then repeated for each combination of locus and variable and is corrected for multiple testing.

The resolution of QTL linkage mapping is generally low (typically ≥ 10 cM) [9]. Thus, a QTL linked to multiple traits may be a single QTL with pleiotropy or different QTLs within the mapping region that affect different traits. Association studies, in contrast, have much higher resolution, and are more feasible for identifying gene pleiotropy. Lange [10] proposed a family-based association method that constructs an overall phenotype by finding a linear combination of traits to maximize heritability. Klei [11] extended this method to population samples. Both methods use principal components, reducing multiple phenotypes to only a single trait, which can cause loss of power. In addition, maximization of heritability and association testing in the same samples may inflate type I error. To address this issue, Klei [11] proposed to split the sample into training and testing data and apply cross-validation to control error inflation, but this further increases computational complexity. In contrast to reduction of phenotypes, direct multivariate regression examines pleiotropy by simultaneous analysis of multiple phenotypes [12].

In this study, we propose to integrate two common methods that test for association by analyzing multiple traits simultaneously: principal components and multivariate regression. However, there are no comprehensive evaluations of this principal-component-based multivariate regression (PCBMR). In our study, we comprehensively evaluated the power and type I error of PCBMR using simulations that varied pleiotropic effects, linkage disequilibrium (LD), proportion of contributed correlation, and number of traits. We also used PCBMR to examine the pleiotropic effects of multiple traits on human abdominal obesity-metabolic syndrome.

Human abdominal obesity-metabolic syndrome [13], a cluster of syndrome phenotypes, increases the risk of developing both diabetes mellitus [14] and cardiovascular disease [15,16]. The prevalence of metabolic syndrome varies with age and sex [17]. Kissebah [18] performed a genome-wide linkage scan with a marker density of 10 cM in 2,209 individuals from 507 Caucasian families. They found one QTL, on chromosome 3q27, that was strongly linked to six phenotypes: body mass index (BMI), waist circumference (WC), hip circumference (HC), weight, insulin, and insulin/glucose (I/G). The results indicated possible pleiotropic effects. Francke replicated this result, finding the same locus on 3q27 through a genome-wide linkage scan of 99 families of northeastern Indian origin [19]. Here, we attempted to identify markers on 3q27 that are associated with the six traits above by using PCBMR to analyze data from the Bogalusa Heart Study [20].

## Results

### Simulation 1, differences in extent of QTL pleiotropic effect

The correlation coefficients between traits *Y_1 _*and *Y_2 _*varied from *-0.35 *to *0.37*, with means for traits increasing as effect *b *increases. PCBMR generated two canonical variables for all simulated data. Power and type I error for the PCBMR and single-trait association studies are summarized in Table 1. When *b = 0*, the QTL had no effect on *Y_1 _*and *Y_2 _*and the type I errors were 4.5%-5.6% for PCBMR, 4.9%-6.1% for single-trait association without Bonferroni adjustment (SATN), and 2.8%-3.0% for single-trait association with Bonferroni adjustment (SATB) for the different models (GEN, ADD, DOM, and REC). Power depends on the assumption of genetic model, with power in decreasing order for ADD, DOM, GEN, and REC. For each model, the following results were obtained: 1) power generally increased in PCBMR, SATN, and SATB as effect *b *got larger, and PCBMR generally had more power than SATB and SATN; 2) the binomial exact test showed that PCBMR was significantly more powerful than SATB for all *b > 0 *(results not shown), and more powerful than SATN for *b > 0.2 *(marked with star); and 3) for b ≤ 0.2, there was no significant power difference between PCBMR and SATN.

**Table 1:** Type 1 error and power of data sets of simulation 1

Effect (b)	PCBMR				Single-Trait Association			
	GEN	ADD	DOM	REC	GEN	ADD	DOM	REC
0	5.1	4.5	5.3	5.6	5.7(2.8)	5.8(3.0)	6.1(2.9)	4.9(3.0)
0.1	5.8	5.4	6.2	4.7	5.6(3.1)	5.8(3.3)	5.9(3.0)	5.3(2.8)
0.2	10.8*	12	11.2	6.8	8.9(4.8)	10.9(6.1)	10.9(5.7)	6.4(3.4)
0.3	14.1*	18.8*	18.3*	9.1*	12.2(8.6)	14.4(9.2)	14.6(9.6)	7.3(4.4)
0.4	21.4*	26.8*	25.2*	11.3	15.9(10.0)	20.5(14.5)	19.9(13.1)	10.4(6.3)
0.5	31.9*	41.9*	36.7*	15.7*	24.3(14.8)	29.1(20.3)	27.3(18.1)	13.6(8.7)
0.6	45.4*	54.9*	50.1*	21.3*	31.6(23.2)	39.9(30.0)	36.1(26.7)	17.2(10.8)
0.7	60.3*	71.4*	65.0*	26.5*	41.9(31.3)	50.5(40.1)	47.2(36.9)	21.6(14.1)
0.8	71.9*	81.9*	77.3*	30.9*	53.3(43.6)	63.6(51.9)	58.2(46.9)	24.2(18.0)
0.9	81.7*	90.8*	84.3*	41.7*	62.5(50.4)	72.7(62.2)	66.1(55.6)	30.4(21.5)
1	91.4*	95.2*	92.8*	48.9*	72.8(62.8)	82.0(73.4)	76.7(67.3)	36.7(27.0)

(The values outside the parentheses are the power (b > 0) or type I error (b = 0) of the single-trait association test without multiple-test adjustment (SATN) and the values inside the parentheses are the power (b > 0) or type I error (b = 0) of the single-trait association test with Bonferroni adjustment (SATB). * indicates that the power of PCBMR is significantly better than that of SATN; GEN: general model without assumption of genetic inheritance; ADD: additive effect model; DOM: dominant model and REC: recessive model)

### Simulation 2, differences in extent of LD between a marker and pleiotropic QTL

Correlation coefficients between *Y_1 _*and *Y_2 _*varied from *-0.25 *to *0.32*, and two canonical variables were generated by PCBMR for pleiotropic association studies. Power and type I error for PCBMR, SATN, and SATB are presented in Table 2. Correlation coefficients (*r*) between tested markers and QTLs ranged from *0 *to *1.0*. A correlation of *r = 0 *indicated that the tested marker and the QTL were independent and that there was no association between them. Under the differing assumptions in different genetic models (GEN, ADD, DOM, and REC), type I error was 4.2%-5.8% for PCBMR, 5.3%-5.8% for SATN, and 2.5%-3.1% for SATB. Power depended on the assumptions of the genetic models, and ADD, DOM, GEN, and REC had powers in decreasing order for all methods. For each model, the following results were obtained: 1) the powers of PCBMR, SATN, and SATB increased as *r *became larger; 2) according to the binomial exact test, PCBMR had significantly greater power than SATB (results not shown) for all *r > 0*, and significantly greater power than SATN when *r > 0.2 *in all but the REC model (marked with star); and 3) for r ≤ 0.2, there was no significant power difference between PCBMR and SATN.

**Table 2:** Type 1 error and power of data sets of simulation 2

LD (r)	PCBMR				Single-Trait Association			
	GEN	ADD	DOM	REC	GEN	ADD	DOM	REC
0	5	4.2	4.6	5.5	5.8(2.5)	5.3(3.1)	5.7(3.1)	5.7(3.1)
0.1	4.1	4.8	5.3	4.7	5.5(2.8)	6.0(3.4)	6.6(3.9)	5.2(2.7)
0.2	9.2	11	10.1	8.2	9.9(5.4)	12.3(6.5)	10.2(6.3)	8.0(4.3)
0.3	14.8	19.6*	16.2*	11.7	14.0(8.1)	17.5(10.5)	14.6(8.4)	10.4(6.2)
0.4	18.7*	24.3*	20.5*	11.7	15.9(10.2)	19.6(11.9)	16.5(10.7)	10.4(6.2)
0.5	26.6*	32.9*	29.7*	11.7	20.1(13.8)	23.8(17.4)	23.4(15.9)	10.4(6.2)
0.6	50.2*	62.8*	56.2*	26.6*	36.6(27.9)	47.0(36.3)	41.4(31.2)	21.2(13.8)
0.7	49.2*	61.6*	56.1*	23.4*	36.7(27.2)	46.8(34.6)	40.6(29.4)	20.7(12.5)
0.8	72.6*	81.5*	76.7*	37.6*	52.0(42.3)	63.1(52.2)	57.2(46.0)	27.9(18.9)
0.9	80.8*	89.7*	86.2*	37.6*	62.0(50.4)	71.9(62.2)	67.9(57.0)	27.9(18.9)
1	91.4*	95.2*	92.8*	48.9*	72.8(62.8)	82.0(73.4)	76.7(67.3)	36.7(27.0)

(See Table 1)

### Simulation 3, trait correlation between effects of two QTL and an environmental variable

The correlation coefficients between simulated traits *Y_1 _*and *Y_2 _*were ≥0.98. Based on this, PCBMR generated a single canonical variable for the pleiotropic association test. The tested QTL exerted a simulated effect *b *from 0 to 4, and based on equation *3*, the percentage of trait correlation contributed by the QTL, *P_ρ_(b)*, ranged from 0 to 20%. The type I error and power related to *P_ρ_(b) *for different methods are summarized in Table 3. A result of *b = 0 *(or *P_ρ_(b) = 0*) indicates that the tested QTL had no pleiotropic effect on the simulated traits. The type I error was 3.9%-5.6% for PCBMR, 4.2%-5.7% for SATN, and 2.4%-3.3% for SATB under the four genetic models, GEN, ADD, DOM, and REC. Power depended on the assumptions of the genetic models, with power in decreasing order for ADD, DOM, GEN, and REC. All methods increased in power as *P_ρ_(b) *increased. When *b = 0.5 *(or *P_ρ_(b) = 0.4%*), power was small for all three analytical methods; *6.4%-8.8% *for PCBMR, *6.1%-8.4% *for SATN, and *3.3%-4.7% *for SATB. When *b *equaled *3.5 *and *4 *(*P_ρ_(b) = 15.7% *and *19.5%*), all methods had power close to *1 *under various genetic models, except recessive ones. For *b > 0*, the binomial exact test showed that PCBMR was not significantly different in power from SATN, but was significantly more powerful than SATB.

**Table 3:** Type 1 error and power of data sets of simulation 3

Effect (b)	PCBMR				Single-Trait Association			
	GEN	ADD	DOM	REC	GEN	ADD	DOM	REC
0	4.7	5	5.6	3.9	5.0(3.2)	5.3(2.7)	5.7(3.3)	4.2(2.4)
0.5	7.6	8.8	7.5	6.4	7.4(4.1)	8.4(4.7)	7.1(4.6)	6.1(3.3)
1	18.1	22.4	22	11.2	17.8(11.2)	22.4(16.2)	21.2(14.1)	11.2(7.2)
1.5	35.3	45.2	40.1	20.3	35.8(24.8)	45.8(34.3)	40.2(28.4)	20.2(13.8)
2	60.4	70.1	66	29.2	60.2(50.3)	70.2(59.8)	66.5(54.9)	28.9(22.0)
2.5	79.2	87.4	82.5	40.8	79.1(70.1)	86.8(79.0)	82.2(75.1)	40.2(30.2)
3	91.2	95.9	93.4	50.6	91.1(85.5)	95.8(91.7)	93.3(88.6)	50.7(40.4)
3.5	97.2	99.3	97.6	62	97.6(94.9)	99.4(98.0)	97.8(96.0)	62.5(50.6)
4	99.6	99.7	99.5	75.4	99.4(98.9)	99.7(99.5)	99.5(99.0)	75.9(63.9)

(See Table 1; Based on equation 3, the percentages of trait correlation contributed by tested QTL are 0%, 0.4%, 1.5%, 3.3%, 5.7%, 8.7%, 12.0%, 15.7% and 19.5% corresponding to b = 0, 0.5, 1, 1.5, 2, 2.5, 3, 3.5, 4)

### Simulation 4, pleiotropic effects on more than two traits

Under this simulation strategy, the number of traits affected by the QTL ranged from 2 to 10. The correlation coefficients between any pair of simulated traits were all ≥0.97 and the expected percentage of correlation contributed by the tested QTL was 8.7%. For all numbers of traits, PCBMR generated one canonical variable for the association test. Results are presented in Table 4. Power depended on genetic model assumptions, with power decreasing in order among ADD, DOM, GEN, and REC. For different numbers of traits and different genetic model assumptions, the power of PCBMR was consistently close to that of SATN, with no significant difference detected by the binomial exact test. Power was approximately equal for different numbers of traits as well. The power of SATB decreased dramatically as the number of traits increased. Compared with SATB, PCBMR had significantly improved power, especially with larger numbers of traits.

**Table 4:** Type 1 error and power of data sets of simulation 4

Traits	PCBMR				Single-Trait Association			
	GEN	ADD	DOM	REC	GEN	ADD	DOM	REC
2	79.1	87.4	82.5	40.8	79.1(70.1)	86.8(79.0)	82.2(75.1)	40.2(30.2)
3	80.6	87.2	83.1	40.2	80.1(67.0)	86.8(75.7)	82.5(70.1)	40.0(25.7)
4	79.4	87.6	83.3	41.2	79.6(62.2)	88.3(72.9)	83.3(65.6)	42.4(24.6)
5	78.6	87.8	83.2	40.9	78.9(59.1)	87.3(69.6)	83.3(63.8)	41.2(18.0)
6	78.4	86.3	83	40.1	78.6(57.2)	86.9(68.4)	82.8(60.7)	40.8(17.9)
7	79.2	86.2	82.4	40.1	78.5(54.6)	86.4(65.7)	82.7(58.5)	40.7(14.6)
8	80.3	85.5	82.4	40.7	80.0(51.6)	86.2(63.3)	82.1(56.0)	40.8(13.7)
9	77.9	85.6	83.1	42.5	78.1(52.3)	86.3(61.5)	82.3(53.9)	42.9(15.3)
10	78.7	87.3	82.6	42.4	78.8(47.4)	87.7(60.4)	83.0(51.4)	42.4(14.7)

(See Table 1)

### Pleiotropic Association Studies of Traits of Abdominal Obesity-Metabolic Syndrome

A total of 1,196 subjects with 5,529 SNPs in the candidate region of chromosome 3 (at 182-227cM or 173.4-198.8 Mb) made up the study population. Quality control measures included the removal of SNPs with minor allele frequencies of ≤0.01 and Hardy-Weinberg equilibrium p-values of ≤1e^-5^, leaving 4,769 SNPs in the study. The characteristics of the study participants are summarized in Table 5 for both males and females, as follows: age (AGE, in years), weight circumference (WEIGHT, in kg), waist circumference (WAIST, in cm), body mass index (BMI, in kg/m^2^), hip circumference (HIP, in cm), plasma insulin level (INSULIN, in μU/mL) and plasma insulin/glucose ratio (I/G). The pairwise correlation coefficients (*r*) among adjusted traits are presented in Table 6. The correlations clustered into two groups, with the first group comprised of WEIGHT, BMI, WAIST, and HIP (r ≥ 0.89) and the second group comprised of INSULIN and I/G (*r = 0.97*).

**Table 5:** Characteristics of study participants

	N	AGE (yrs)	WEIGHT (kg)	WAIST (cm)	BMI (kg/m^2^)	HIP (cm)	INSULIN (μU/mL)	I/G
Male	517	36.2 (4.4)	91.8 (20.6)	98.4 (15.9)	29.1 (6.2)	107.6 (11.6)	12.8 (9.6)	0.14 (0.09)
Female	679	35.7 (4.6)	78.8 (22.2)	89.3 (17.7)	29.5 (8.0)	110.2 (15.7)	13.2 (14.7)	0.15 (0.16)

Mean (standard deviation)

**Table 6:** Pair-wise correlation coefficients, r, between adjusted traits

	WEIGHT	BMI	WAIST	HIP	INSULIN	I/G
**WEIGHT**	1.00					
**BMI**	**0.95**	1.00				
**WAIST**	***0.93***	***0.91***	1.00			
**HIP**	***0.93***	***0.92***	***0.89***	1.00		
**INSULIN**	0.46	0.46	0.47	0.41	1.00	
**I/G**	0.43	0.43	0.44	0.38	***0.97***	1.00

**Figure 1:**
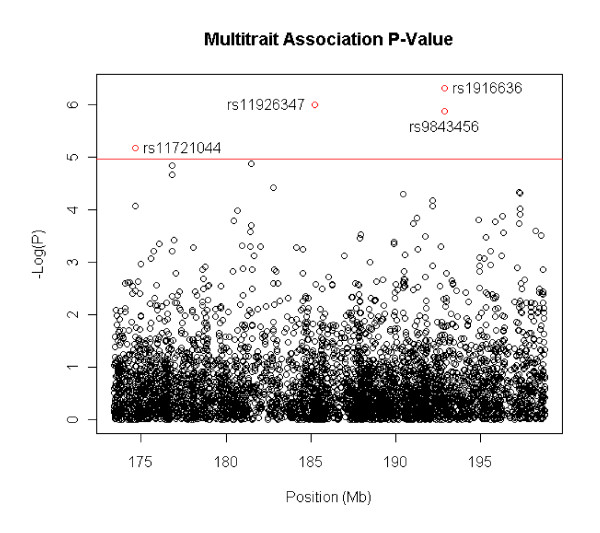
**Pleiotropic association study of WEIGHT, HIP, BMI and WAIST based on general model by PCMBA on the candidate region, 182-227cM of Chromosome 3. **There are 4769 total SNPs. The x axis is the SNP position and y axis is negative logarithm of p-value, i.e. -log (P).

**Figure 2:**
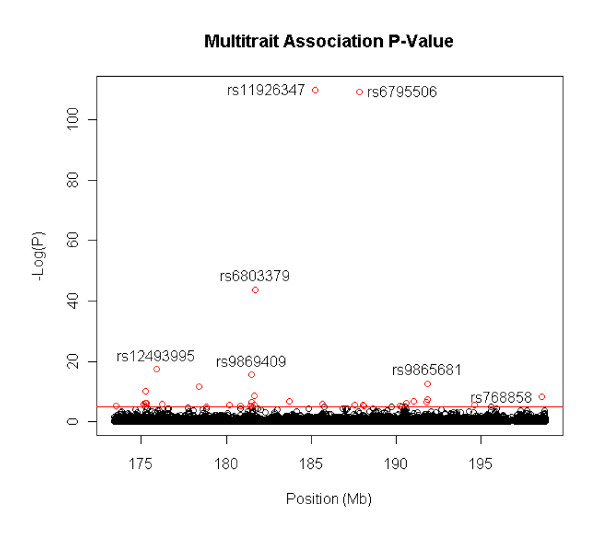
**Pleiotropic association study of INSULIN and I/G by PCMBA based on general model on the candidate region, 182-227cM of Chromosome 3. **There are total 4769 SNPs. The x axis is the SNP position and y axis is negative logarithm of p-value, i.e. -log (P).

The results of the PCBMR pleiotropic association studies based on the GEN model are presented in Figures 1 and 2. Markers with significant p-values (≤1e^-5^) are summarized in Tables 7 and 8. For these markers, analyses based on recessive, dominant and additive models were conducted, and the best genetic model and its p-value were documented.

**Table 7:** Significant pleiotropic association with WEIGHT, HIP, BMI and WAIST

SNP	-Log(P)	POSITION	Function
rs11721044	5.19(5.88^1^)	174.64	NLGN1 (intron)
rs11926347	6.00(6.46^1^)	185.21	ABCC5 (intron)
rs9843456	5.88(6.70^3^)	192.85	
rs1916636	6.33(7.15^3^)	192.85	

Position is in megabase. The smallest p-value and its corresponding genetic model, additive (1), dominant (2) or (3) recessive, are enclosed inside parenthesis.

**Table 8:** Significant pleiotropic association with INSULIN and I/G

SNP	POSITION	-LOG(P)	Function
rs669552	173.54	5.20(5.79^3^)	FNDC3B (intron)
rs6786075	175.12	5.76(6.56^3^)	NLGN1 (intron)
rs9854235	175.25	6.21(6.73^3^)	NLGN1 (intron)
rs6445137	175.26	10.03(10.75^3^)	NLGN1 (intron)
rs6798572	175.32	6.01	NLGN1 (intron)
rs12493995	175.89	17.34(18.29^3^)	
rs9878945	176.22	5.80(6.21^3^)	NAALADL2 (intron)
rs9809218	176.56	7.17	NAALADL2 (intron)
rs11920602	178.39	11.59 (12.40^3^)	TBL1XR1 (Intron)
rs17633881	178.83	5.05(5.72^3^)	
rs6797848	180.17	5.63(6.36^3^)	
rs7611854	180.17	5.63(6.36^3^)	
rs11927983	180.82	5.09(5.89^1^)	NDUFB5 (Intron)
rs4854964	181.40	5.15(5.87^3^)	
rs1525276	181.43	5.13(5.75^3^)	
rs7643438	181.47	6.36 (7.18^3^)	
rs9869409	181.48	15.44(16.28^3^)	
rs7647526	181.63	5.45	
rs7650795	181.67	8.55(9.11^3^)	
rs6803379	181.68	43.76(44.14^3^)	
rs11926347	185.21	109.86(110.37^3^)	ABCC5 (intron)
rs6798973	185.67	5.73(6.55^3^)	
rs6786711	187.56	5.52(6.32^3^)	DGKG (intron)
rs6795506	187.81	109.05(110.37^3^)	AHSG (near gene 5')
rs2082940	188.06	5.50(6.30^3^)	ADIPOQ (utr 3')
rs7628649	188.07	5.25(6.06^3^)	
rs16863863	190.20	5.36(6.11^3^)	
rs7614680	190.57	6.06(6.81^3^)	
rs1515495	191.00	6.81	TP63 (intron)
rs4571225	191.81	6.44(7.05^3^)	IL1RAP (intron)
rs9821331	191.81	7.43(8.32^3^)	IL1RAP (intron)
rs9865681	191.83	12.63(13.59^3^)	IL1RAP (intron)
rs902192	194.60	5.66(6.41^3^)	
rs768858	198.56	8.15(8.87^3^)	

Refer to Table 7

For the first trait group of WEIGHT, BMI, WAIST, and HIP, PCBMR generated a single canonical variable that explained 94.1% of the variance. With Bonferroni adjustment, PCBMR using the GEN model found four SNPs with significant pleiotropic association (p <*1e^-5^*) (Figure 1). Among these, SNP rs11721044 at 174.6 Mb and rs11926347 at 185.2 Mb were located in genes NLGN1 (OMIM 600568) and ABCC5 (OMIM 60521), respectively (Table 7).

For the second trait group of INSULIN and I/G, PCBMR also generated a single canonical variable, and this variable explained 98.6% of the variance. Using the GEN model, thirty-four SNPs passed Bonferroni significance level (Figure 2), of which 17 were found within 11 genes. SNP rs11926347, in an intron of ABCC5 (OMIM 60521), and SNP rs6795506, near the 5' end of AHSG (OMIM 138680), had extremely small p-values (Table 8). Among the other nine genes, ADIPOQ (OMIM 605441, 612556) has been widely reported to be associated with obesity and diabetes [21-24]; FNDC3B (OMIM 611909) is involved in positive regulation of adipogenesis [25]; and DGKG (OMIM 601854) and AHSG (OMIM 138680) have been reported to be associated with obesity-related metabolic traits [26,27]. The remaining genes have no reported relation to obesity-related metabolic traits based on our literature review.

SNP rs11926347 in ABCC5 showed significant pleiotropic association in both groups and the p-value was extremely small in the second group of traits (*-log(P) = 109.86*). To validate these PCBMR results, this SNP was extracted for further study. The SNP's phenotype distribution, divided by genotype, is presented in Table 9. Its alleles are '*A*' and '*G*' and the frequency of the minor allele *'A' *is *0.02*. The Hardy-Weinberg Equilibrium (HWE) exact test[28] yielded a p-value of 0.37. Homozygotes for the minor allele ('A/A') exhibited only one extreme mean value for all six traits. Heterozygotes ('G/A') had smaller values than 'A/A' homozygotes but much larger values than homozygotes for the major allele ('G/G'). SATN analyses with adjustment for age and sex gave p-values of ≤1.15*10^-5 ^for all traits (results not shown). With allele A as a reference, we conducted an examination of pleiotropic effects for rs11926347 based on additive, dominant, and recessive models. The corresponding -log_10_(P) was 6.46, 5.92, and 2.12 for the first group's traits and 11.65, 4.74, and 110.37 for the second group's traits for additive, dominant, and recessive models, respectively. These results indicate that the additive model best suits the first trait group's association and the recessive model best suits the second trait group's association. After dropping the single homozygote, analyses based on different genetic models generated the same results. The significant association was absent in the second group's traits (-log_10_(p) = 1.14), but still present in the first group's traits (-log_10_(p) = 5.2). These results indicated that allele '*A*' may be involved in pleiotropic association with metabolic syndrome and merits attention and inclusion in genetic studies of obesity in the future.

**Table 9:** Summary of rs11926347 in ABCC5

	A/A	G/A	G/G
Frequency	1	45	1150
AGE (yrs)	26.5	36.06(5.32)	35.97(4.43)
Male% (kg)	0	0.49	0.43
BMI (kg/m2)	48.08	34.50(10.26)	29.11(7.03)
WEIGHT (kg)	144.8	99.33(30.03)	83.79(21.83)
WAIST(cm)	129.1	103.3(22.5)	92.8(17.2)
HIP (cm)	146.27	117.23(19.63)	108.74(13.79)
INSULIN (μU/mL)	247	16.11(12.04)	12.69(10.68)
IG	2.68	0.17(0.12)	0.15(0.11)

(HWE: p-value = 0.37)

## Discussion

Most current association studies have been based on single trait-single marker or single trait-multiple marker tests. These kinds of studies lose power in identifying genes with pleiotropic effects. In some cases, genes with pleiotropy may be found by separately testing each trait. However, two major issues make this strategy not always appropriate. First, pleiotropic effects for each trait may be too weak to be identified. Second, multiple testing problems may either lower the power or inflate the type I error. It is therefore important to develop methods that can test for association by analyzing multiple traits simultaneously.

In this paper, we present the use of PCBMR as a method which detects pleiotropic effects by combining principal component methods and multivariate regression. PCBMR generates a set of independent canonical variables based on principal components. Each canonical variable is associated with multiple traits and the sum of all variables explains at least 80% of the variation. Analysis of canonical variables is simultaneously implemented by multivariate regression. The statistic of PCBMR is simply the sum of individual test statistics. PCBMR is computationally efficient and can be easily implemented by most statistical packages. This makes PCBMR fast and feasible not only for candidate-gene association studies but also for genome-wide association studies (GWAS).

Comprehensive studies of simulated data have shown that PCBMR has well-controlled type I error, about 5%, when a tested marker has no pleiotropy (simulation 1 and 3) or exhibits linkage equilibrium to the pleiotropic QTL, in the case of pleiotropic tested markers (simulation 2). The power of PCBMR depends on the extent of the pleiotropic effect and on the LD of the QTL. Larger pleiotropic effects and higher LD result in larger power (simulation 1 and 2). When the trait correlation caused by pleiotropy was not strong (simulation 1), the number of canonical variables was the same as the number of traits and the power was reasonably high, even compared with SATN. When there were strong correlations among traits (simulation 4), the reduced number of variables resulted in fewer degrees of freedom for the PCBMR test, and the power of PCBMR was as high as SATN. However, SATN always has much higher type I error than PCBMR due to multiple testing. PCBMR was robust to conflicting effects from environmental factors or other, untested QTLs (simulation 3). In all cases, multi-trait association analyses using PCBMR were much more powerful than multiple single-trait association analyses using SATB. For all tests, multiple traits simultaneously studied by PCBMR were compared with the single trait with the best power as determined by SATN and SATB. The present study showed that PCBMR is at least as powerful as SATN and more powerful than SATB under pleiotropy.

PCBMR has great extensibility. For equations (*1*) and (*2*), PCBMR can be extended to any distribution in the exponential family and the parameter *θ *can take any link function (e.g. logistic or log) that relates a mean, *Z_i, _*to covariates [29]. The covariate can be a single variable for one marker or multiple variables for different markers. In addition, non-genetic factors with or without interaction terms can also serve as the covariate. The final statistic, approximating the χ^2 ^distribution, is again the simple sum of statistics from separate regressions of a canonical variable on single or multiple covariates.

Comparisons of power estimates among PCBMR, SATB, and SATN in this study were based on analyses of the simulated additive model. To verify these findings, we tried studies on both simulated dominant and recessive models, and the same conclusions were obtained - that pleiotropic association studies by PCBMR are more powerful than single-trait association studies by either SATN or SATB (results not shown here). In addition, influences of model mismatch were also observed. For example, we observed that a pleiotropic study based on an additive model sacrificed its power when the true model was dominant or recessive. In addition, we observed that all studies based on the general model have acceptable power. In contrast to the additive model, which assumes linear trends of genotypic effect, and the dominant and recessive models, which assume equal effects of two genotypes for an SNP, the general model aims to separately estimate the effect of each genotype without any restriction. Therefore, PCBMR based on the general model has the advantage of testing for a pleiotropic effect when a complex trait has no obvious Mendelian inheritance.

As a real example, PCBMR was applied to test association in a study of traits-weight, waist circumference, BMI, hip circumference, plasma insulin, and insulin-glucose ratios-of abdominal obesity-metabolic syndrome in the Bogalusa Heart Study cohort. The traits were clustered into two groups based on two previously identified linkage peaks [18] and these two groups exhibited strong correlation. After multiple-test adjustment, PCBMR successfully identified several SNPs associated with the traits, especially in the trait group of INSULIN and I/G. Some of the genes had been well-characterized in prior studies, e.g. FNDC3B, which is involved in adipogenesis [25]. However, the functions of most of the genes were not yet explicitly clear at the time of the analysis. For example, some genes (e.g. ABCC5) are known to be related to energy metabolism, but are they truly involved in obesity-metabolic syndrome? If they are, what are their functions? The results from the use of PCBMR in this study offer guidance for future researchers in understanding genetic mechanisms and pathways in the pathogenesis of this human disease.

Although this study illustrates many advantages of PCBMR, there are also some challenges to be faced in terms of practical application. In contrast to pleiotropic linkage studies that map a QTL to a large locus [30], PCBMR-based studies can provide a higher resolution QTL position. However, the association may not justify the true pleiotropy of the identified marker or gene. For example, when PCBMR identifies a significant association by studying multiple traits, we may not observe significant association with a particular trait. This may result from either a weak pleiotropic effect or no effect at all. Such differentiation is generally difficult to achieve by statistical analysis. Further experimental studies or repeated studies with larger sample size are therefore necessary to confirm that the association is due to pleiotropy. In addition, the power of PCBMR depends on the assumptions of the genetic model, and misuse of a model will decrease power. The number of canonical variables also depends on the threshold. A value of 0.8 is used in simulation studies to explain at least 80% of the variation. Although this threshold is widely accepted for principal component analysis and has been proven to be suitable in our simulation studies, the ideal threshold may depend on practical data, with the exact value generally not known in advance. Furthermore, pleiotropic association is based on canonical variables, and to get an exact estimate of the effect on an original trait, a reverse transformation needs to be conducted.

Another challenge is to decide which traits should be studied simultaneously by PCBMR. Some strategies may help to address this challenge. Candidate traits could be those related to each other in the same pathway leading to a disease or symptom. For example, greater weight and BMI are correlated with obesity. Candidate traits could also include traits with linkage to the same region, such as two groups of traits with linkage peaks in two separate loci, as found in our studies of abdominal obesity-metabolic syndrome. Nevertheless, it is possible that two traits without much correlation may be strongly affected by a common gene. For example, in our simulation *1*, though the effect is strong at *b = 1*, the correlation coefficient (r) ranges from -0.35 to 0.37 with a mean of only about 0.10. In this case, selection of traits mainly depends on currently established knowledge.

PCA is an important tool for data mining that transforms a larger number of correlated variables into a smaller number of independent variables, *i.e.*, principal components. Factor analysis (FA), another important analytical tool, identifies common factors that capture variance-covariance of multiple variables with random error. PCA, in contrast, identifies principle components, with the restriction that random error must be zero[31]. Therefore, FA could be better suited to the analysis of observed traits with measured errors and to tests of genetic pleiotropy in some cases. The PCA-based multivariate regression proposed in this study can be easily extended to FA-based regression for testing of genetic pleiotropy in these cases. This can be implemented by replacing principal components with common factors. However, without estimation of random error, PCA is more computationally efficient for analyses involving large amounts of genetic data, and has great advantages in terms of practical application[32]. For most cases, PCA and FA procedures typically yield highly similar results[32]. This was also the case in the present study; we conducted an additional FA-based multivariate regression analysis of pleiotropic association with metabolic traits, and the results were the same as those obtained by PCBMR (please see additional file 1). This is consistent with previous findings that PCA and FA behave similarly in tests of genetic pleiotropy[33].

In spite of its potential challenges, PCBMR is a powerful and computationally efficient method of studying the huge amounts of genetic data generated by advanced technology, *e.g. *GWAS. For a large number of markers, we suggest a strategy of traditional single-trait studies on a candidate marker that PCBMR declares significant. This strategy can not only help to explain PCBMR results, but also has great advantages over traditional single-trait studies in alleviating multiple testing problems. Suppose there are *N *markers and *m *traits, and the experimental type I error is controlled at *α*. The significance level for tests of a marker in traditional single-trait studies is α/(N*M). This level is extremely small when both *N *and *M *are large. In contrast, for a candidate marker, the significance level for this strategy is *α/(N+M)*. Generally, for most association studies and GWAS, *M *is much smaller than *N*, and the significance level will approximate *α/N*.

## Conclusion

In summary, we propose the use of PCBMR, a computationally efficient method for the testing of gene pleiotropy. Although PCBMR is a combination of two established methods- principal components and multivariate regression-we are the first to comprehensively evaluate this technique in its combined form. The simulation studies described here indicate that this method is powerful for different kinds of pleiotropy. In spite of some challenges for its use in practical studies, PCBMR can greatly increase the power of association studies under pleiotropy and can broaden understanding of a gene's functions as well as its pathway and mechanisms. PCBMR is not only a useful method for candidate-gene based studies; as the generation of high-throughput expression data becomes increasingly efficient, PCBMR can be used to study pleiotropy in analyses of massive amounts of data, such as GWAS.

## Methods

### Principal Component Based Multivariate Regression (PCBMR)

Given a set of traits, PCBMR uses the method of principal component analysis (PCA) [34,35] to construct one or more independent canonical variables based on a specific threshold (*θ*). Suppose Y = (*Y_1_, Y_2_,..., Y_m_) *represents variables of *m *traits. PCA searches for *k *principal components (*k ≤ m*), which is a new *k*-dimensional coordinate system. Within each principal component a canonical variable is generated as a linear combination of the original *m *traits with maximized variance. The search can be simplified by using the decomposition of the covariance of *Y*. However, different units of trait measures may result in different decompositions. To overcome this issue, PCBMR standardizes original traits with mean 0 and sample variance 1. The standardized variable (*Y^s^*) for trait *Y *is generalized by:

$$Y^{S}={\left(V^{1/2}\right)}^{-1}(Y-\mu ),$$

where *μ *is the mean of *Y *and *V *is a diagonal matrix with diagonal items equal to the variances of the corresponding traits. For *Y^S^*, *Cov*(*Y*^*S*^) = (*V*^1/2^)^-1^*C*ov(*Y*)(*V*^1/2^)^-1 ^= *ρ*, so its covariance and correlation matrices are the same and ρ = ΓΛΓ^T^, where Γ is the matrix of eigenvectors and Λ is the diagonal matrix of eigenvalues.

PCA finds the weighting vector *δ = (δ^1^, ..., δ^p^)^T ^*that maximizes the variance of canonical variable *z = δ^T^Y^S ^*[36]. This can be expressed by:

$$Var(z)=\underset{\left\{\delta :||\delta ||=1\right\}}{\max}Var(\delta^{T}Y^{s})=\underset{\left\{\delta :||\delta ||=1\right\}}{\max}\delta^{T}\rho \delta .$$

*δ *is proved to be an eigenvector of *ρ *[36]. If we use *z = [z_1_, z_2_, ..., z_m_]^T ^*representing *m *canonical variables, then *z = Γ^T^Y *and Var(z) = Λ. The correlation between *z_i _*and *Y_j_^S ^*is (Γ_ij_Λ_jj_)^1/2^, and the sum of squares of correlations between all *m *canonical variances and any original trait is equal to *1*, i.e. $\sum_{i=1}^{m}\rho (z_{i},Y_{j}^{S})=1$[36]. Therefore, a canonical variable *z_i _*can explain a fraction of the variance for each *Y_j_^S^*, and any maker associated with *z_i _*will indicate association with the original traits. Canonical variables with very low eigenvalues explain only a minuscule fraction of the variance of the original traits and can be deleted from the analysis [37]. PCBMR chooses the first k principal components to construct canonical variables that explain over 80% of variation.

Suppose *z_1_, z_2_, ...,z_k _*have normal distributions with mean *μ_i _*and variance *σ_i_^2 ^*(*i = 1,2,...,k*). Since all canonical variables are mutually independent, their joint distribution that takes the general form of the exponential family is:

(1) $$\begin{array}{l} f(z_{1},z_{2},...,z_{k}|\theta_{1},\theta_{1},...,\theta_{k};\phi_{1},\phi_{1},...,\phi_{k})\\ \begin{array}{cccc} & & & =\prod_{i=1}^{k}\exp[\frac{z_{i}\theta_{i}-b(\theta_{i})}{a(\phi_{i})}+c(z_{i},\phi_{i})] \end{array} \end{array}$$

Where *θ_i _= μ_i_, ϕ_i _= σ_i_^2^, a(ϕ_i_) = ϕ_i_, b(θ_i_) = θ_i_^2^/2 *and *c(z_i_, ϕ_i_) = -[z_i_^2^/ϕ_i_+log(2πϕ_i_)]/2 *[29].

In multivariate regression, PCBMR takes the canonic link. The mean regression model is *μ_i _= Xβ_i_+Wτ_i_*, where *X *and *W *are explanatory variables of tested markers and other controlled variables, respectively, and *β_i _*and *τ_i _*are their corresponding parameter vectors, which denoting effects on the μ, of the *i-*th canonical variable. The null hypothesis of no association (*H_0_*) is:

$$\beta_{1}=\beta_{2}=...=\beta_{k}=0$$

We define the full model as the one without restriction of *H_0 _*and the nested model as the one with restriction of *H_0_*. PCBMR uses the likelihood ratio test (LRT) for the goodness of fit between full and nested models. Suppose *z_ij _*is the observed canonical variable *z_i _*on *j*th subject (*j = 1,2,...,N*). The sample likelihood *L(θ) *based on equation (1) is:

(2) $$\begin{array}{l} L(\theta )=L(\theta_{1},\theta_{2},...,\theta_{k}|\{z_{ij}\})\\ =\prod_{i=1}^{k}\left\{\prod_{j=1}^{N}\exp[\frac{z_{ij}\theta_{i}-b(\theta_{i})}{a(\phi_{i})}+c(z_{ij},\phi_{i})]\right\} \end{array}$$

The LRT statistic T is -2[logL($\tilde{\theta }$) - logL($\hat{\theta }$)], where $\tilde{\theta }$ is the maximum likelihood estimate (MLE) of θ for the nested model and the $\hat{\theta }$ MLE of θ for the full model. When the mean regression model, *θ_i _= μ_i _= Xβ_i_+Wτ_i_*, is input into equation (2), the T statistic is simplified to:

$$T=\sum_{i=1}^{k}(\frac{\sum_{j=1}^{N}{\left(z_{ij}-{\hat{\mu }}_{i}\right)}^{2}-\sum_{j=1}^{N}{\left(z_{ij}-{\tilde{\mu }}_{i}\right)}^{2}}{{\hat{\phi }}_{i}})=\sum_{i=1}^{k}T_{i}$$

The mean estimates, ${\hat{\mu }}_{i}$ and, ${\tilde{\mu }}_{i}$ are calculated by simple linear regression of *z_i _*on [X W] and W respectively. $\sum_{j=1}^{N}{\left(z_{ij}-{\hat{\mu }}_{i}\right)}^{2}$ and $\sum_{j=1}^{N}{\left(z_{ij}-{\tilde{\mu }}_{i}\right)}^{2}$ are deviances of the full and nested models, respectively, and ${\hat{\phi }}_{i}={\hat{\sigma }}_{i}^{2}$ is the estimate of dispersion, all of which can be calculated by almost all statistical packages. *T_i _*is the χ^2 ^distributed LRT statistic for testing marker association with canonical trait *z_i _*by simple linear regression. The sum of *T_i _*also has a χ^2 ^distribution with degrees of freedom equal to the difference of parameter numbers between the full and the nested model. A large T causing rejection of *H_0 _*indicates at least one *β*_*i *_≠ 0 and the presence of association attributable to the pleiotropic effects of multiple markers.

### Simulation Studies

The power of PCBMR may depend on many factors; some of these are: 1) the extent of the QTL pleiotropic effect; 2) the extent of LD between the tested marker and the pleiotropic QTL; 3) the portion of the trait correlation contributed by the tested QTL relative to the portion contributed by other QTL and environmental factors; and 4) the number of traits in the study. For each simulation, 1,000 datasets were generated. Type I error and power were calculated as percentages of the datasets, with *p-*value ≤ 0.05. Without loss of generality, in the following design, the QTL is simulated with additive effects on different traits. *Y_1_, Y_2_, ...Y_k _*are original QTL traits, *U_1_, U_2_, ..., U_k _*are the population means of *K *traits, *X *is the genotype of pleiotropic QTL denoted by *0*, *1 *and *2*, *b_1_*, *b_2_*,..., *b_k _*are additive effects, and *E_1_, E_2_, ..., E_k _*are random errors.

#### Simulation 1, different extents of pleiotropic effects in QTL

The minor allele frequency of QTL is *0.2 *(*p = 0.2*), and simple linear regression models, *Y_1 _*= *U_1_*+X*b_1_+ *E_1 _*and *Y_2 _*= *U_2_*+X*b_2_+ *E_2_*, are used to simulate traits *Y_1 _*and *Y_2_*. To simplify the simulation, we set *U_1 _*= 0 and *U_2 _*= 50, *E_1 _*and *E_2 _*to a normal distribution of mean **0 **and standard deviation *2 *(*E_1_~E_2_~N(0, 2^2^)*), and *b_1 _= b_2 _= b *with *11 *different effects from *0 *to *1.0 *with steps of *0.1*.

#### Simulation 2, different extents of LD between a marker and a pleiotropic QTL

In this situation, the QTL (*p_1 _= 0.2*) is not known directly. Instead, a marker of minor allele frequency *0.2 *(*q_1 _= 0.2*) with LD to the QTL is genotyped for the test. Linear regression models, *U_1_*, *U_2_*, *E_1_*, and *E_2 _*are set as above. The additive effects of *b_1 _*and *b_2 _*are fixed at 1. LD was measured using a correlation coefficient (*r*) set between *0 *and *1 *with steps of *0.1*. For a pair of alleles of a tested marker, denoted *A_1 _*and *A_2_*, and those of the QTL, denoted *B_1 _*and *B_2_*, the following equation was used to calculate the joint allele frequencies of the tested marker and QTL. Based on *r*, *D *is calculated as $r*\sqrt{p_{1}(1-p_{1})q_{1}(1-q_{1})}$, and the joint allele frequencies of the tested marker and QTL are calculated as *f(A_1_B_1_) = p_1_q_1_+D*, *f(A_1_B_2_) = p_1_(1-q_1_)-D*, *f(A_2_B_1_) = (1-p_1_)q_1_-D *and *f(A_2_B_2_) = (1-p_1_)(1-q_1_)+D *[38]. Assuming Hardy-Weinberg Equilibrium (HWE) for both QTL and marker, we can infer frequencies of the tested marker genotypes for simulation, given the frequency of QTL genotypes, *f(AiAj|Bi'Bj') (i, i', j, j' = 1,2)*.

#### Simulation 3, trait correlation based on the effects of two QTL and an environmental variable

Two linear regression models, *Y_1 _*= *U_1_*+X*b_1_+Q*c_1_+W*d_1_+E_1 _and *Y_2 _*= *U_2_*+X*b_2_+Q*c_2_+W*d_2_+ *E_2_*, were used to simulate traits *Y_1 _*and *Y_2_*, where *U_1 _*= 0, *U_2 _*= 50, and *E_1_~E_2_~N(0, 0.5^2^)*. The effects of *b_1 _= b_2 _= b *are from *0 *to *4 *with steps of *0.5*. *Q *is the second QTL with pleiotropic effects *c_1 _*= *c_2 _*= 4. Both *X *and *Q *have minor allele frequencies of 0.2. *W *is an environmental covariate with a standard normal distribution *N(0, 1) *and effects *d_1 _*= *d_2 _*= 4. The correlation, ρ(*Y_1_*, *Y_2_*), between *Y_1 _*and *Y_2_*is:

$$\begin{array}{l} \rho (Y_{1},Y_{2})=\frac{Cov(Y_{1},Y_{2})}{\sqrt{\mathrm{var}(Y_{1})}\sqrt{\mathrm{var}(Y_{2})}}\\ =\frac{b_{1}b_{2}\mathrm{var}(X)+c_{1}c_{2}\mathrm{var}(Q)+d_{1}d_{2}\mathrm{var}(W)}{\sqrt{\mathrm{var}(Y_{1})}\sqrt{\mathrm{var}(Y_{2})}} \end{array}$$

The proportion of the correlation contributed by QTL *X*, *P_ρ_(b)*, is

(3) $$\begin{array}{l} P_{\rho }(b)=\frac{b_{1}b_{2}\mathrm{var}(X)}{b_{1}b_{2}\mathrm{var}(X)+c_{1}c_{2}\mathrm{var}(Q)+d_{1}d_{2}\mathrm{var}(W)}\\ =\frac{0.32b^{2}}{0.32b^{2}+21.12} \end{array},$$

so *P_ρ_(b) *increases as *b *increases.

#### Simulation 4, pleiotropic effects on more than two traits

Based on the linear regression model, *Y_i _*= *U_i_*+X*b+Q*c+W*d+E (*i = 1,2, ...10*), 2 to 10 traits were separately simulated in each dataset. Without loss of generality, *X*, *Q*, and *W *were defined as above with *b = 2.5*, *c = 4*, and *d = 4 *set correspondingly. *E *is distributed as normal, *N(0, 0.5^2^)*, and U_i _= (i-1)*50 for *i = 1, 2,..., 10*.

Power and type I error were estimated for PCBMR under the four simulation conditions. For comparison, we conducted single-trait association studies using classical linear regression with (STAB) and without (SATN) Bonferroni adjustment. For single-trait association studies, only the trait with the largest power or type I error was presented in the paper. Based on different assumptions of the genetic models, there are four possible ways of processing the *X *variable for genotypes, which take values *0*, *1 *and *2*: 1) *X *is treated as a factor with three levels for the general model (GEN) without assumption of any genetic inheritance; 2) *X *is a linear variable in the additive model (ADD); 3) *X *is 0 for genotypes *0 *and *1*, and is *1 *for genotype *2 *in the dominant model (DOM); and 4) *X *is *0 *for genotype *1 *and is *1 *for genotypes *1 *and *2 *in the recessive model (REC). All four assumptions were considered separately for association tests by PCBMR and single trait regression.

### Power comparison by binomial exact test

Without loss of generality, we created indicator variables *M_1 _*and *M_2 _*for methods 1 and 2, respectively, where method 1 is PCBMR and method 2 is either SATB or SATN. The value of the variables was *1 *for a significant *p-*value and *0 *otherwise. Matched pairs of *M_1 _*and *M_2 _*were tested by the binomial exact test [39], based on the fact that *Σ_i_M_1i_|(Σ_i_M_1i_+Σ_i_M_2i_) = N_m _*has binomial distribution (*N_m_*, *p*), *i = 1, 2, ....1000 *for the simulated data above. For *Σ_i_M_1i _> N_m_/2*, the null and alternative hypotheses are *p ≤ 0.5 *and *p > 0.5 *respectively. Rejection of the null hypothesis indicates that method *1 *is significantly more powerful than method *2*. For *Σ_i_M_1i _< N_m_/2*, the null and alternative hypotheses are *p ≥ 0.5 *and *p < 0.5*, respectively. Rejection of the null hypothesis indicates that method *1 *is significantly less powerful than method *2*. To strictly evaluate the power of PCBMR, we compared it to method *2 *for the trait with the largest power.

### Pleiotropic Association Studies of Abdominal Obesity-Metabolic Syndrome

We applied PCBMR to search for markers associated with multiple traits related to abdominal obesity-metabolic syndrome in the Bogalusa Heart Study, a community-based investigation of the evolution of cardiovascular disease risk beginning in childhood [20]. Based on previous studies [18], we focused our studies on six traits (body mass index (BMI), waist circumference (WAIST), hip circumference (HIP), weight (WEIGHT), insulin (INSULIN) and insulin/glucose (I/G)) and on chromosome 3 from 182-227 cM (173.4-198.8 Mb), which contains potential pleiotropic QTL [18,19]. The most recent measures were used for all subjects. SNP genotyping was performed using data from Illumina Human610 BeadChips. Only SNPs passing our quality control measures were included in the study. BMI, WAIST, HIP and WEIGHT traits have a linkage peak at 189-190 cM, and insulin and I/G at 202-203 cM [18]. Hence, associations with the multiple traits of BMI, WAIST, HIP, and WEIGHT and of INSULIN and I/G were separately studied by PCBMR. These traits may depend on sex and age. Instead of analyzing original traits directly, traits were regressed by sex and age according to the following formula: Y_i _= U+AGE*b_1_+AGE^2^*b_2_+SEX+E_i_, where residuals (*E_i_*) were used as adjusted traits for association studies by PCBMR. The inheritance model for markers underlying abdominal obesity-metabolic syndrome is generally not known before pleiotropic association tests. We thus applied a general model that estimates the effect of each possible genotype for an SNP association in the sample. After susceptibility markers were identified, different models (additive, dominant and recessive) with the minor allele as reference were also examined in the comparisons. The p-value was adjusted for multiple tests by the Bonferroni method. The number of SNPs in the pleiotropic study was 4,769, so the significance level for testing an SNP association was 1e^-5^.

## Authors' contributions

HM developed and implemented the method. HM and WC performed the simulations, analysis and interpretation of the data. All authors participated in planning and discussion of the study. All authors read and approved the final manuscript.

## Supplementary Material

Additional file 1 **Factor analysis-based study of pleiotropic association. **Table of significant pleiotropic association and figure of p-values of SNPs in linkage region. Click here for file

## Electronic-Database Information

Online Mendelian Inheritance in Man (OMIM), http://www.ncbi.nlm.nih.gov/Omim/

## References

[B1] Jiang C Zeng ZB Multiple trait analysis of genetic mapping for quantitative trait loci Genetics 1995 140 3 1111 1127 7672582 10.1093/genetics/140.3.1111 PMC1206666

[B2] Korol AB Ronin YI Kirzhner VM Interval mapping of quantitative trait loci employing correlated trait complexes Genetics 1995 140 3 1137 1147 7672584 10.1093/genetics/140.3.1137 PMC1206668

[B3] Mangin B Thoquet P Grimsley N Pleiotropic QTL analysis Biometrics 1998 54 88 99 10.2307/2533998

[B4] Calinski T Kaczmarek Z Krajewski P Frova C Sari-Gorla M A multivariate approach to the problem of QTL localization Heredity 2000 84 Pt 3 303 310 10.1046/j.1365-2540.2000.00675.x 10866532

[B5] Hackett CA Meyer RC Thomas WT Multi-trait QTL mapping in barley using multivariate regression Genet Res 2001 77 1 95 106 10.1017/S0016672300004869 11279835

[B6] Knott SA Haley CS Multitrait least squares for quantitative trait loci detection Genetics 2000 156 2 899 911 11014835 10.1093/genetics/156.2.899 PMC1461263

[B7] Korol AB Ronin YI Nevo E Hayes PM Multi-interval mapping of correlated trait complexes Heredity 1998 80 3 273 284 10.1046/j.1365-2540.1998.00253.x

[B8] Weller JI Wiggans GR Vanraden PM Ron M Application of a canonical transformation to detection of quantitative trait loci with the aid of genetic markers in a multi-trait experiment Theor App Genet 1996 92 998 1002 10.1007/BF00224040 24166627

[B9] Mackay TF The genetic architecture of quantitative traits Annu Rev Genet 2001 35 303 339 10.1146/annurev.genet.35.102401.090633 11700286

[B10] Lange C van Steen K Andrew T Lyon H DeMeo DL Raby B Murphy A Silverman EK MacGregor A Weiss ST A family-based association test for repeatedly measured quantitative traits adjusting for unknown environmental and/or polygenic effects Stat Appl Genet Mol Biol 2004 3 Article17 16646795 10.2202/1544-6115.1067

[B11] Klei L Luca D Devlin B Roeder K Pleiotropy and principal components of heritability combine to increase power for association analysis Genet Epidemiol 2008 32 1 9 19 10.1002/gepi.20257 17922480

[B12] Stich B Piepho HP Schulz B Melchinger AE Multi-trait association mapping in sugar beet (Beta vulgaris L.) Theor Appl Genet 2008 117 6 947 954 10.1007/s00122-008-0834-z 18651127

[B13] Bjorntorp P Metabolic implications of body fat distribution Diabetes Care 1991 14 12 1132 1143 10.2337/diacare.14.12.1132 1773700

[B14] Haffner SM Valdez RA Hazuda HP Mitchell BD Morales PA Stern MP Prospective analysis of the insulin-resistance syndrome (syndrome X) Diabetes 1992 41 6 715 722 10.2337/diabetes.41.6.715 1587398

[B15] Isomaa B Almgren P Tuomi T Forsen B Lahti K Nissen M Taskinen MR Groop L Cardiovascular morbidity and mortality associated with the metabolic syndrome Diabetes Care 2001 24 4 683 689 10.2337/diacare.24.4.683 11315831

[B16] Srinivasan SR Myers L Berenson GS Changes in metabolic syndrome variables since childhood in prehypertensive and hypertensive subjects: the Bogalusa Heart Study Hypertension 2006 48 1 33 39 10.1161/01.HYP.0000226410.11198.f4 16769996

[B17] Esposito K Pontillo A Giugliano F Giugliano G Marfella R Nicoletti G Giugliano D Association of low interleukin-10 levels with the metabolic syndrome in obese women J Clin Endocrinol Metab 2003 88 3 1055 1058 10.1210/jc.2002-021437 12629085

[B18] Kissebah AH Sonnenberg GE Myklebust J Goldstein M Broman K James RG Marks JA Krakower GR Jacob HJ Weber J Quantitative trait loci on chromosomes 3 and 17 influence phenotypes of the metabolic syndrome Proc Natl Acad Sci USA 2000 97 26 14478 14483 10.1073/pnas.97.26.14478 11121050 PMC18944

[B19] Francke S Manraj M Lacquemant C Lecoeur C Lepretre F Passa P Hebe A Corset L Yan SL Lahmidi S A genome-wide scan for coronary heart disease suggests in Indo-Mauritians a susceptibility locus on chromosome 16p13 and replicates linkage with the metabolic syndrome on 3q27 Hum Mol Genet 2001 10 24 2751 2765 10.1093/hmg/10.24.2751 11734540

[B20] Pickoff AS Berenson GS Schlant RC Introduction to the symposium celebrating the Bogalusa Heart Study Am J Med Sci 1995 310 Suppl 1 S1 2 7503110 10.1097/00000441-199512000-00001

[B21] Vasseur F Helbecque N Dina C Lobbens S Delannoy V Gaget S Boutin P Vaxillaire M Lepretre F Dupont S Single-nucleotide polymorphism haplotypes in the both proximal promoter and exon 3 of the APM1 gene modulate adipocyte-secreted adiponectin hormone levels and contribute to the genetic risk for type 2 diabetes in French Caucasians Hum Mol Genet 2002 11 21 2607 2614 10.1093/hmg/11.21.2607 12354786

[B22] Filippi E Sentinelli F Trischitta V Romeo S Arca M Leonetti F Di Mario U Baroni MG Association of the human adiponectin gene and insulin resistance Eur J Hum Genet 2004 12 3 199 205 10.1038/sj.ejhg.5201120 14673476

[B23] Menzaghi C Ercolino T Di Paola R Berg AH Warram JH Scherer PE Trischitta V Doria A A haplotype at the adiponectin locus is associated with obesity and other features of the insulin resistance syndrome Diabetes 2002 51 7 2306 2312 10.2337/diabetes.51.7.2306 12086965

[B24] Vimaleswaran KS Radha V Ramya K Babu HN Savitha N Roopa V Monalisa D Deepa R Ghosh S Majumder PP A novel association of a polymorphism in the first intron of adiponectin gene with type 2 diabetes, obesity and hypoadiponectinemia in Asian Indians Hum Genet 2008 123 6 599 605 10.1007/s00439-008-0506-8 18465144

[B25] Tominaga K Kondo C Johmura Y Nishizuka M Imagawa M The novel gene fad104, containing a fibronectin type III domain, has a significant role in adipogenesis FEBS Lett 2004 577 1-2 49 54 10.1016/j.febslet.2004.09.062 15527760

[B26] Thorleifsson G Walters GB Gudbjartsson DF Steinthorsdottir V Sulem P Helgadottir A Styrkarsdottir U Gretarsdottir S Thorlacius S Jonsdottir I Genome-wide association yields new sequence variants at seven loci that associate with measures of obesity Nat Genet 2009 41 1 18 24 10.1038/ng.274 19079260

[B27] Andersen G Burgdorf KS Sparso T Borch-Johnsen K Jorgensen T Hansen T Pedersen O AHSG tag single nucleotide polymorphisms associate with type 2 diabetes and dyslipidemia: studies of metabolic traits in 7,683 white Danish subjects Diabetes 2008 57 5 1427 1432 10.2337/db07-0558 18316360

[B28] Emigh TH A Comparison of Tests for Hardy-Weinberg Equilibrium Biometrics 1980 36 4 627 642 10.2307/2556115 25856832

[B29] Faraway JJ Extending Linear Models with R: Generalized Linear, Mixed Effects and Nonparametric Regression Models 2006 Boca Raton: Chapman & Hall/CRC

[B30] Gardner KM Latta RG Shared quantitative trait loci underlying the genetic correlation between continuous traits Mol Ecol 2007 16 20 4195 4209 10.1111/j.1365-294X.2007.03499.x 17850272

[B31] Larose DT Data mining methods and models 2006 Hoboken, New Jersey: John Wiley & Sons, Inc

[B32] Velicer WF Jackson DN Component Analysis versus Common Factor Analysis: Some Issues in Selecting an Appropriate Procedure Multivariate Behavioral Research 1990 25 1 28 10.1207/s15327906mbr2501_1 26741964

[B33] Wang X Kammerer CM Anderson S Lu J Feingold E A comparison of principal component analysis and factor analysis strategies for uncovering pleiotropic factors Genet Epidemiol 2009 33 4 325 331 10.1002/gepi.20384 19048641 PMC3042259

[B34] Hotelling H Analysis of a complex of statistical variables into principal components Journal of Educational Psychology 1933 24 417 441 10.1037/h0071325

[B35] Jolliffe IT Principal Component Analysis 2002 2 New York: Springer

[B36] Härdle W Simar L Applied Multivariate Statistical Analysis 2007 2 New York: Springer

[B37] Mardia KV Kent JT Bibby JM Multivariate Analysis 1979 London: Academic Press

[B38] Weir BS Genetic Data Analysis 2: Methods for Discrete Population Genetic Data 1996 2 Sinauer Associates, Sunderland, MA

[B39] Agresti A Categorical Data Analysis 2002 2 New Jersey.: John Wiley & Sons, Inc

