# Supplementary material for: Principal-component-based multivariate regression for genetic association studies of metabolic syndrome components
Source: BMC Genet. 2010 Nov 9;11:100. doi: 10.1186/1471-2156-11-100 (PMC2991276; doi:10.1186/1471-2156-11-100)

**Additional File**

Factor analysis (FA) generally requires at least three variables to get a stable common factor. To compare with PC-based study, we conducted FA-based multivariate regression analysis of pleiotropic association in the first group comprised of WEIGHT, BMI, WAIST, and HIP. The results of significant SNPs and p-values of all SNPs were presented at supplemental table 1 and figure 1.

**Table 1: s**ignificant pleiotropic association

| **SNP** | **-Log(P)** | **POSITION** | **Function** |
| --- | --- | --- | --- |
| rs11721044 | 5.28(5.94^1^) | 174.64 | NLGN1 (intron) |
| rs11926347 | 6.14(6.56^1^) | 185.21 | ABCC5 (intron) |
| rs9843456 | 5.94(6.75^3^) | 192.85 |  |
| rs1916636 | 6.38(7.21^3^) | 192.85 |  |

Position is in megabase. The smallest p-value and its corresponding genetic model, additive (1), dominant (2) or (3) recessive, are enclosed inside parenthesis.

**Figure 1**: Pleiotropic association study of WEIGHT, HIP, BMI and WAIST based on general model by FA-based multivariate regression analysis on the candidate region, 182-227cM of Chromosome 3. There are 4769 total SNPs. The x axis is the SNP position and y axis is negative logarithm of p-value, i.e. –log (P).


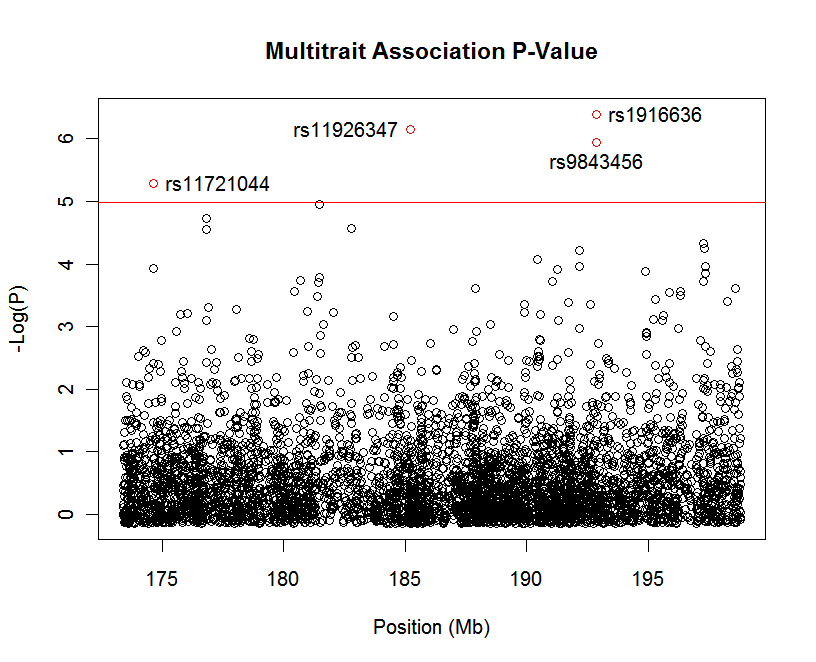

Supplement: Additional file 1 — Factor analysis-based study of pleiotropic association. Table of significant pleiotropic association and figure of p-values of SNPs in linkage region. [file 1471-2156-11-100-S1.DOC]
